# Supplementary material for: Acute Downregulation but Not Genetic Ablation of Murine MCU Impairs Suppressive Capacity of Regulatory CD4 T Cells
Source: Int J Mol Sci. 2023 Apr 24;24(9):7772. doi: 10.3390/ijms24097772 (PMC10178810; doi:10.3390/ijms24097772)
Supplement: Supplementary file 1 [file ijms-24-07772-s001.zip › ijms-2319545-supplementary.pdf]

Supplementary Figure S1

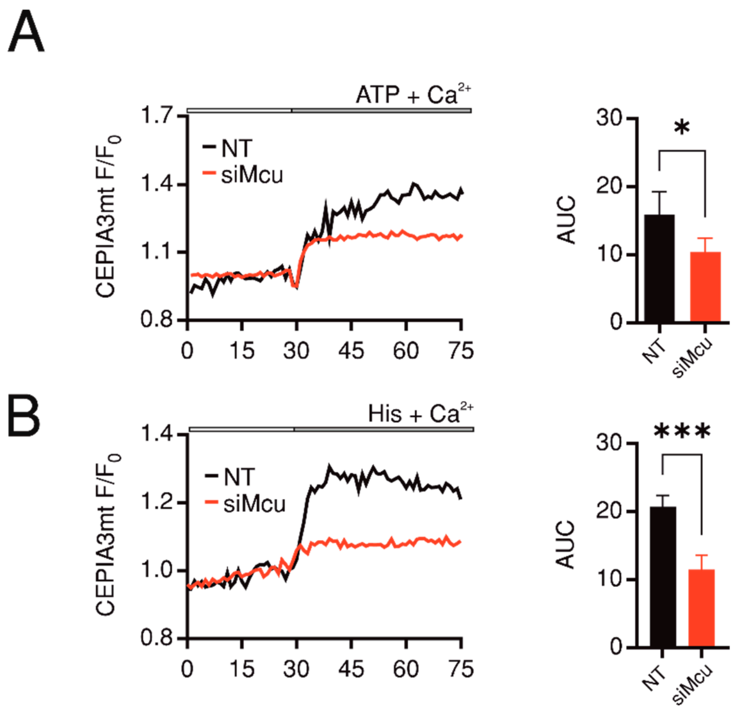

Figure S1. Mcu downregulation reduces mitochondrial Ca^{2+} uptake. (**A**) Representative images showing fluorescence of mtCEPIA3 corresponding to mitochondrial Ca^{2+} levels before (basal) and after application of 2 mM ATP in EL-4 murine T cell line transfected with non-targeting (NT) or Mcu silencing (siMcu) RNA; Average traces showing changes of fluorescence ratio of mtCEPIA3 over time in cells transfected as in (A) where the mitochondrial Ca^{2+} uptake is triggered by application of 2 mM ATP (**B**) or 100 µM Histamine (**C**). Bar graphs represent averages ± S.E.M of area under influx curve (AUC) from 3 independent experiments, and (32-48) cells per condition, * $p < 0.05$, *** $p < 0.001$. 48h before the RNA transfection cells were transduced with viral particles encoding mtCEPIA3.
